# Focally perfused succinate potentiates brain metabolism in head injury patients

**DOI:** 10.1177/0271678X16672665

**Published:** 2016-01-01

**Authors:** Ibrahim Jalloh, Adel Helmy, Duncan J Howe, Richard J Shannon, Peter Grice, Andrew Mason, Clare N Gallagher, Matthew G Stovell, Susan van der Heide, Michael P Murphy, John D Pickard, David K Menon, T Adrian Carpenter, Peter J Hutchinson, Keri LH Carpenter

**Affiliations:** 1 Division of Neurosurgery, Department of Clinical Neurosciences, University of Cambridge, UK; 2 Department of Chemistry, University of Cambridge, UK; 3 Wolfson Brain Imaging Centre, Department of Clinical Neurosciences, University of Cambridge, UK; 4 Division of Neurosurgery, Department of Clinical Neurosciences, University of Calgary, Canada; 5 MRC Mitochondrial Biology Unit, Cambridge, UK; 6 Division of Anaesthesia, Department of Medicine, University of Cambridge, UK

**Keywords:** Traumatic brain injury (human), microdialysis, nuclear magnetic resonance spectroscopy, cerebral metabolism, succinate

## Abstract

Following traumatic brain injury, complex cerebral energy perturbations occur. Correlating with unfavourable outcome, high brain extracellular lactate/pyruvate ratio suggests hypoxic metabolism and/or mitochondrial dysfunction. We investigated whether focal administration of succinate, a tricarboxylic acid cycle intermediate interacting directly with the mitochondrial electron transport chain, could improve cerebral metabolism. Microdialysis perfused disodium 2,3-^13^C_2_ succinate (12 mmol/L) for 24 h into nine sedated traumatic brain injury patients' brains, with simultaneous microdialysate collection for ISCUS analysis of energy metabolism biomarkers (nine patients) and nuclear magnetic resonance of ^13^C-labelled metabolites (six patients). Metabolites 2,3-^13^C_2_ malate and 2,3-^13^C_2_ glutamine indicated tricarboxylic acid cycle metabolism, and 2,3-^13^C_2_ lactate suggested tricarboxylic acid cycle spinout of pyruvate (by malic enzyme or phosphoenolpyruvate carboxykinase and pyruvate kinase), then lactate dehydrogenase-mediated conversion to lactate. Versus baseline, succinate perfusion significantly decreased lactate/pyruvate ratio (p = 0.015), mean difference −12%, due to increased pyruvate concentration (+17%); lactate changed little (−3%); concentrations decreased for glutamate (−43%) (p = 0.018) and glucose (−15%) (p = 0.038). Lower lactate/pyruvate ratio suggests better redox status: cytosolic NADH recycled to NAD^+^ by mitochondrial shuttles (malate-aspartate and/or glycerol 3-phosphate), diminishing lactate dehydrogenase-mediated pyruvate-to-lactate conversion, and lowering glutamate. Glucose decrease suggests improved utilisation. Direct tricarboxylic acid cycle supplementation with 2,3-^13^C_2_ succinate improved human traumatic brain injury brain chemistry, indicated by biomarkers and ^13^C-labelling patterns in metabolites.

## Introduction

After traumatic brain injury (TBI), complex pathology follows, intertwining intracranial dynamics, electrophysiological responses and cerebral metabolism.^1–4^ Despite modern advances, many TBI survivors experience long-term disability. Better neurocritical care requires better understanding of post-injury brain pathophysiology. Early studies focussed on ischaemia, nowadays minimised by protocol-driven therapy maintaining adequate cerebral perfusion with intracranial pressure below a critical threshold.^5^

Despite seemingly adequate provision of metabolic fuels and oxygen, the injured brain sometimes cannot utilise them efficiently: termed ‘mitochondrial dysfunction’, the exact basis is not understood. One potentially relevant process is the reduced Ca^2+^ uptake state after TBI,^6^ which will depress the activities of several mitochondrial dehydrogenases,^7^ leading to suboptimal mitochondrial metabolism. Mitochondria are the cells’ ‘powerhouses’ performing high-yielding energy metabolism. Glycolysis in the cytoplasm produces pyruvate, taken up by mitochondria and processed via the tricarboxylic acid (TCA) cycle, which interacts with the mitochondrial electron transport chain (ETC; also termed respiratory chain) driving ATP synthase. The ETC requires molecular oxygen as terminal electron acceptor. Correlating with unfavourable clinical outcome, high brain extracellular lactate/pyruvate ratio (LPR) suggests high glycolytic activity^3^: glucose converted to pyruvate generating a low ATP yield, then lactate dehydrogenase (LDH) converts pyruvate plus NADH to lactate plus NAD^+^, allowing further glycolysis.

Succinate is energetically important – a TCA cycle intermediate and a direct contact between the TCA cycle and the mitochondrial ETC. Succinate conversion to fumarate in the TCA cycle is by ETC complex II (succinate dehydrogenase (SDH)), on the mitochondrial inner membrane. Soluble mitochondrial enzymes perform all the other TCA cycle steps; the NADH produced interacts with ETC complex I, then electron transport runs sequentially (missing out complex II) to Coenzyme Q (CoQ; also termed ubiquinone), complex III, Cytochrome C and complex IV.^8^ Succinate, contrastingly, misses out complex I, and electron transport runs sequentially from complex II to CoQ, complex III, Cytochrome C and complex IV with molecular oxygen as terminal electron acceptor, culminating in conversion of oxygen to water.^8^ Complexes I, III and IV export protons across the mitochondrial inner membrane creating a proton electrochemical potential gradient, driving ATP synthesis at complex V (ATP synthase), converting ADP to ATP.^8^

Succinate can support compromised mitochondria, as it bypasses complex I that can be more vulnerable to inhibition/damage than complexes II, III and IV.^9^ Optimization of residual energy-producing ability may prevent the ATP level from dropping critically low initiating cell death pathways. ‘Bypassing’ defective ETC components sustained muscle ATP synthesis in a patient with genetically defective complex III, using vitamins C and K.^10^ In experimental sepsis (which involves energy dysfunction), succinate infusion prevented fall in liver ATP content,^11^ and prolonged survival.^12^

**Figure 1.**
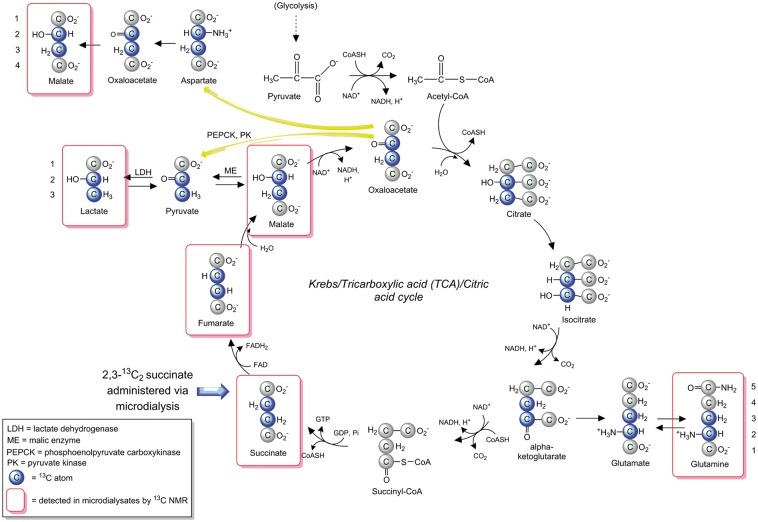
Schematic of metabolism of 2,3-^13^C_2_ succinate via the TCA cycle and spin-out pathways. Blue-filled circles indicate ^13^C atoms. Red rectangular outlines indicate metabolites detected by ^13^C NMR in microdialysates. For further details, see Results and Discussion sections. ME: malic enzyme; PC: pyruvate carboxylase; PDH: pyruvate dehydrogenase; PEPCK: phosphoenolpyruvate carboxykinase; PK: pyruvate kinase.

Succinate uptake is via the SLC13 family of Na^+^-coupled di-carboxylate and tri-carboxylate transporters.^13–15^ SLC13 occur widely, including in brain, astrocytes and neurons where succinate uptake and metabolism were also shown using radio-labelling.^16–18^ Additionally, nonspecific uptake might occur in any cells with increased plasma membrane permeability. Succinate is metabolised to fumarate by SDH, ETC complex II on the inner mitochondrial membrane. SDH occurs at high concentrations only within mitochondria.^19^ Hence, appearance of metabolites with characteristic ^13^C nuclear magnetic resonance (NMR) doublet signals will clearly indicate uptake and mitochondrial metabolism of 2,3-^13^C_2_ succinate. For a schematic of labelling via the TCA cycle, see Figure 1. In the TCA cycle, succinate’s metabolite fumarate is converted to malate and then to oxaloacetate (OAA) that can proceed in the TCA cycle to alpha-ketoglutarate, and then spin out (via glutamate) to glutamine. Also, OAA can spin out aspartate, which can exit mitochondria (via the glutamate/aspartate carrier) via the malate-aspartate shuttle mechanism.^20^ In the cytosol, aspartate is converted to OAA and thence to malate, which can re-enter mitochondria (via the malate/alpha-ketoglutarate carrier) and/or may emerge from the TCA cycle via this pathway.

**Figure 2.**
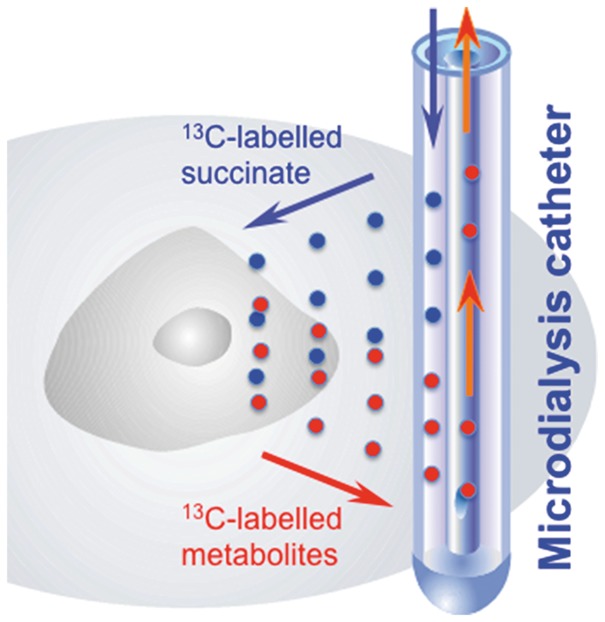
Schematic of ^13^C-labelled microdialysis. Microdialysis is used both to deliver ^13^C-labelled succinate focally into the brain extracellular space and simultaneously collect those metabolite molecules that exit from the cells. Source: Adapted from Carpenter et al.^23^ ©2014 The Authors. Published by Elsevier B.V. Open access under a CC–BY licence.

We tested the hypothesis that 2,3-^13^C_2_ succinate (disodium salt) administered by cerebral microdialysis would fuel and enhance the TCA cycle and improve cerebral metabolism focally in TBI patients. We analysed metabolites using NMR of the emerging microdialysates. We successfully used ^13^C-labelled microdialysis (Figure 2) previously with different substrates.^21–23^ Microdialysates were also analysed for conventional clinical biomarkers of brain chemistry,^3^ at baseline and during succinate perfusion. Double ^13^C-labelling proves unambiguously that substrate molecules crossed from the perfusate into the brain extracellular space, entered cells and were metabolised, exported into the extracellular fluid, and recovered by the microdialysis catheter.

## Patients and methods

### Patients

The protocol was approved by the National Research Ethics Service (NRES) Committee East of England – Cambridge Central (REC Reference No. 11/EE/0463). Written, informed consent was obtained from the next of kin for all subjects. The study was carried out in conformation with the spirit and the letter of the Declaration of Helsinki 1975 (and revised in 1983). Patients (age over 16 years) with severe TBI, defined as cranial trauma with consistent CT scan findings and a post-resuscitation Glasgow Coma Scale ≤ 8 were recruited. Patients were treated according to local TBI management protocols, including endotracheal intubation, ventilation, sedation, muscular paralysis and maintenance of blood sugar (serum glucose) concentration within the target range 4–7 mmol/L.^5^

### Microdialysis technique

CMA 71 microdialysis catheters (membrane 10 mm, cut-off 100 kDa) (M Dialysis AB, Stockholm, Sweden) were placed via a triple lumen cranial access device (Technicam, Newton Abbot, UK) into frontal lobe. For patients with diffuse injury, the right frontal region was chosen. If one hemisphere was more injured than the other, the more injured hemisphere was monitored. Catheters were placed neither into, nor adjacent to, lesions identified on neuroimaging, e.g. contusions. Licox brain tissue oxygen concentration (PbtO_2_) monitoring probes (Integra LifeSciences Corporation, Plainsboro, NJ) were inserted alongside the microdialysis catheters via the same cranial access device.

Catheters were perfused with CNS Perfusion Fluid (M Dialysis AB), composed of NaCl (147 mmol/L), KCl (2.7 mmol/L), CaCl_2_ (1.2 mmol/L) and MgCl_2_ (0.85 mmol/L) in water supplemented with 12 mmol/L disodium 2,3-^13^C_2_ succinate (isotopic enrichment 99%, chemical purity 99%) from Cambridge Isotope Laboratories, Inc (Tewksbury, MA) and formulated in CNS perfusion fluid by the Manufacturing Unit, Department of Pharmacy, Ipswich Hospital NHS Trust (Ipswich, UK), who then tested the formulations for purity, sterility, freedom from endotoxins and absence of pyrogenicity, before release for use in patients. The 12 mmol/L concentration was chosen for 2,3-^13^C_2_ succinate, because it represents the same number of carbon atoms (12 mmol/L × 4 carbon atoms per succinate molecule = 48 mmol of carbon atoms/L) equivalent to glucose at the upper end^24^ of its microdialysate concentration range (8 mmol/L × 6 carbon atoms per glucose molecule = 48 mmol of carbon atoms/L). Glucose concentrations of 8 mmol/L in microdialysates are rare; we deliberately chose this high level as being above the more frequently seen levels (which are usually under 5 mmol/L) to ensure that there was no substrate limitation and we could augment the TCA cycle effectively. Moreover, with microdialysis delivery of a substrate, a diffusion gradient will exist such that the tissue levels achieved will not exceed the perfusate concentration.

Microdialysate collection vials were changed and analysed hourly on a bedside ISCUS analyser (M Dialysis AB) employing enzymatic colorimetric assays for glucose, lactate, pyruvate, glutamate and glycerol, during perfusion with 2,3-^13^C_2_-succinate, and for a baseline perfusion period with unsupplemented CNS perfusion fluid. The latter was either before or after the 2,3-^13^C_2_ succinate perfusion period, with a 2 h exclusion margin applied to the ISCUS data analysis to allow for run-in or wash-out. By testing standard solutions of the five ISCUS analytes at concentrations representative of those in vivo, in CNS-perfusion fluid, with and without disodium succinate standard (12 mmol/L), we established that succinate did not interfere with the ISCUS enzymatic colorimetric chemistry.

### NMR analysis

Brain microdialysate samples analysed on the ISCUS at the bedside were then stored at 4℃ (or –80℃ if storage for longer than a few days was necessary) prior to NMR analysis. Microdialysate samples from a 24-h period during 2,3-^13^C_2_ succinate perfusion were pooled for each individual patient, due to sensitivity limitations of NMR equipment (see ‘Limitations’ subsection of the Discussion for further explanation). For NMR analysis, 20 µL of deuterium oxide (D_2_O) and 50 µL internal reference standard from a stock solution of 24.0 mmol/L 3 -(trimethylsilyl)-1-propanesulfonic acid sodium salt (also termed 2,2-dimethyl-2-silapentane-5-sulfonate sodium salt or 4,4-dimethyl-4-silapentane-1-sulfonate sodium salt; DSS) (Sigma-Aldrich, Gillingham, UK) in CNS perfusion fluid was added to 180 µL of the pooled microdialysate sample, then transferred into 3 mm NMR tubes (Hilgenberg GmbH, Malsfeld, Germany).

^13^C and ^1^H NMR spectra were acquired on a Bruker Avance III HD 500 MHz spectrometer (Bruker BioSpin GmbH, Karlsruhe, Germany) with a dual ^1^H/^13^C cryoprobe (CP DUL500C/H, Bruker BioSpin GmbH), and TopSpin software (Bruker GmbH). For further details of NMR measurements, see Jalloh et al.^22^ Direct-observe ^13^C NMR is a wide-ranging screening technology not requiring operators to make prior decisions of what to seek. NMR does not involve physical contact with the sample, which is enclosed in an individual glass tube throughout measurement, eliminating cross-contamination or sample carry-over.

Fractional enrichment (%) is defined as 100 × [^13^C]/([^13^C] + [^12^C]) where square brackets indicate concentrations. [^13^C] was determined from the ^13^C NMR spectra and [^12^C] for lactate from the ^1^H spectra, using the calibration principles described previously.^22^ Total glutamine concentration (^12^C and ^13^C) was quantified by reverse-phase high-performance liquid chromatography.^25^ The natural abundance of ^13^C is 1.1% of all carbon atoms, and ^13^C results presented for lactate and glutamine were expressed after subtracting this natural background from the ^13^C singlet NMR signals. ^13^C doublet NMR signals for lactate and glutamine were not background-subtracted, because the probability of two natural endogenous ^13^C atoms being next to each other is 1.1% × 1.1% = 0.01%.

### Statistical analysis

Changes between baseline and succinate perfusion periods were assessed using Wilcoxon signed rank test (StatView version 5, SAS Institute, Cary, NC), with p < 0.05 for significance. The corresponding effect sizes (matched-pairs rank-biserial correlation r) were calculated by the method described by Kerby.^26^

## Results

### Demography

Nine severe TBI patients (seven male, two female) aged 18–60 years (median 47 years) were studied using 2,3-^13^C_2_ succinate (disodium salt; 12 mmol/L) perfused via the microdialysis catheter, and for a baseline period with plain unsupplemented perfusion fluid (without 2,3-^13^C_2_ succinate). Table 1 describes demography, catheter positions and PbtO_2_ measured alongside microdialysis in 7/9 of the patients. Median PbtO_2_ measurements were above 20 mmHg in five patients and above 15 mmHg in the other two patients.

**Table 1.** Patient demography.

TBI Patient i.d. number	Age (years)	Sex	Injury mechanism	GCS at scene/15	2,3-^13^C_2_ succinate perfusion period start time (hours from injury)	Marshall grade (admission)	Catheter location^a^	CT description	PbtO_2_ at baseline and during 2,3-^13^C_2_ succinate perfusion (mmHg)
1	56	F	Fall	10	26.0	6a	L frontal	EDH, contusions	25.3, 31.1
2	52	M	RTC	4	121.3	3	R frontal	DAI	–
3	18	M	RTC	8	41.9	3	R frontal	DAI, contusions	21.8, 20.4
4	47	M	RTC	8	36.9	2b	R frontal	DAI, contusions	19.9, 27.4
5	41	F	Fall	3	67.5	6b	R frontal	R ASDH	–, 35.7
6	20	M	Assault	14	83.0	3	R frontal	DAI, contusions	–, 15.8
7	60	M	Fall	11	103.9	2d	R frontal	DAI, contusions	18.6, 17.9
8	47	M	RTC	9	160.4	2b	R frontal	DAI	–
9	46	M	RTC	6	79.5	3	R frontal	L ASDH	34.9, 34.9

M: male; F: female; RTC: road traffic collision; GCS: Glasgow Coma Scale score; L: left; R: right; EDH: extradural haematoma; DAI: diffuse axonal injury; ASDH: acute subdural haematoma; PbtO_2_: brain tissue oxygen tension (median values; – not measured).

a Catheter (microdialysis) location: for patients with a diffuse injury, the right frontal region was chosen; if there was greater injury to one hemisphere rather than the other, the side with the greater burden of injury was monitored. Catheters were inserted via a cranial access device and were placed neither into nor adjacent to lesions (such as contusions) identified on neuroimaging. PbtO_2_ monitoring probes were inserted alongside the microdialysis catheters via the same cranial access device.

### Bedside analysis results

**Figure 3.**
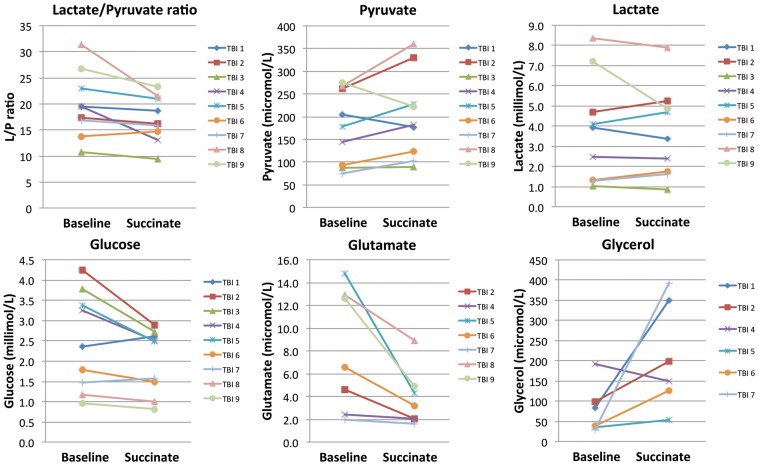
ISCUS clinical microdialysis analyser measurements. Results are during 24 h baseline perfusion (with plain unsupplemented CNS perfusion fluid) and during 24 h perfusion with 2,3-^13^C_2_ succinate (disodium salt; 12 mmol/L). Symbols joined by lines represent individual patients (TBI Patients 1–9). Each pair of data-points indicates median levels at baseline and during succinate perfusion, respectively, for that patient. Note that, for Patients 6 and 7, the lactate concentrations were similar (but not identical), so their symbols and lines are very close to each other. For Patients 1 and 2, the baseline period was post-succinate, while for the other seven patients, the baseline period was pre-succinate. Changes between baseline and succinate perfusion were significant for lactate/pyruvate ratio (p = 0.0152), glucose (p = 0.038) and glutamate (p = 0.018) by Wilcoxon’s signed rank test.

Microdialysate measurements (ISCUS analyser) of glucose, lactate, pyruvate, glutamate, glycerol and LPR are shown in Figure 3 and Supplementary Table 1. The ISCUS cannot measure other species. ISCUS measurements were acquired for the nine TBI patients for a 24-h period while the microdialysis catheter was perfused with plain unsupplemented CNS perfusion fluid directly before or after a 24-h perfusion period with 2,3-^13^C_2_ succinate (12 mmol/L). Each data-point shown is the median concentration for that patient during the relevant 24 h period (24 × 1 h vials). Paired (baseline vs. succinate) data were available in all nine patients for glucose, lactate, pyruvate and LPR, and in 7/9 patients for glutamate and 6/9 patients for glycerol. We chose pair-wise statistics (Wilcoxon signed rank test) for comparing baseline vs. succinate perfusion, so that each patient was represented by a pair of data-points preserving data connectivity within each patient. This avoided risk of bias, rather than pooling data and using unpaired statistics that would have incorrectly assumed too many degrees of freedom and over-estimated significance. In addition to Wilcoxon signed rank test significance p values, we also report effect size (matched-pairs rank-biserial correlation r).^26^

Versus baseline, succinate perfusion significantly decreased the LPR (p = 0.015; effect size r = 0.91), decreasing in 8/9 patients and increasing in 1/9, mean difference −12%. This was due to increased pyruvate concentration, increasing in 7/9 and decreasing in 2/9, mean difference + 17%, missing significance (p = 0.11; effect size r = 0.60). Lactate concentration changed insignificantly (p = 0.86; effect size r = 0.07), increasing in 4/9 and decreasing in 5/9, mean difference −3%. Glycerol concentration change did not reach significance (p = 0.075; effect size r = 0.81), increasing in 5/6 and decreasing in 1/6. Glutamate concentration decreased significantly (p = 0.018; effect size r = 1.00) in 7/7, mean difference−43%. Glucose also decreased significantly (p = 0.038; effect size r = 0.78), decreasing in 7/9 and increasing in 2/9, mean difference −15%. The greatest decreases in microdialysate glucose were in the four individuals (ranging from −22.9% to −31.7%) whose microdialysate glucose baseline concentration was above 3 mmol/L. In the two patients with the highest baseline LPRs of 31.3 and 26.8, these decreased to 21.5 and 23.3, respectively, during succinate perfusion, thus bringing them below 25, regarded as a crucial threshold.^3,27^ These results are graphed in Figure 3 and tabulated in Supplementary Table 1.

Changes in ISCUS data are modest rather than extreme, as we micro-dosed a focal region of brain. While we cannot rule out some spontaneous changes in brain chemistry, a much larger unsupplemented microdialysis TBI study showed no clear longitudinal trends.^24^ Comparing changes *within* patient (baseline vs. succinate supplementation) was preferable to comparing pooled data for the supplemented patients with a separate control unsupplemented group, as is well known that there is much variation between patients for ISCUS data. Using each patient as his/her own control was logical in this small group.

### NMR results

**Figure 4.**
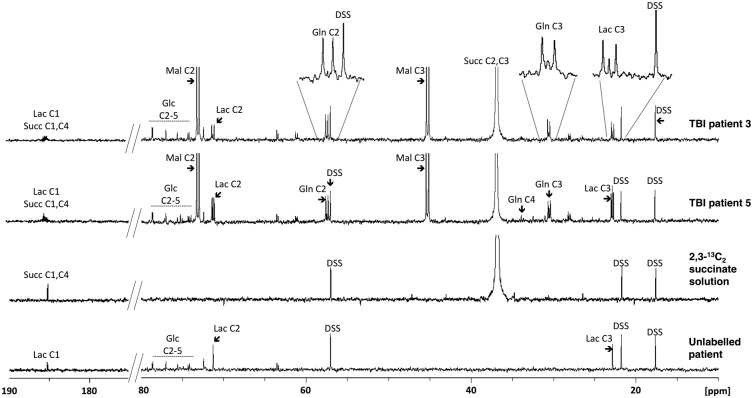
Illustrative examples of ^13^C NMR spectra. Upper two spectra are for microdialysates from TBI Patients 3 and 5 who received perfusion with 2,3-^13^C_2_ succinate disodium salt (12 mmol/L). For comparison, the 2,3-^13^C_2_ succinate solution (before perfusion) (third spectrum) and microdialysate from an unlabelled TBI patient (using plain unsupplemented CNS perfusion fluid) (fourth spectrum) are also shown. Glc: glucose; Lac: lactate; Gln: glutamine; Mal: malate; DSS: 4,4-dimethyl-4-silapentane-1-sulfonate sodium salt (the internal reference standard). Spectra were run from −20 ppm to + 250 ppm. The main reference DSS signal at 0 ppm, and fumarate (138 ppm singlet for equivalent C2 and C3, in the spectra of patients with 2,3-^13^C_2_ succinate perfusion) are not shown in the ranges illustrated.

NMR analysis of brain microdialysates was performed for six TBI patients who received 2,3-^13^C_2_ succinate microdialysis perfusion (Figure 4 illustrates examples). To achieve this, 24 × 1 h microdialysate vials were pooled for each patient, due to sensitivity limitations of our NMR equipment (see ‘Limitations’ subsection of the Discussion for further explanation). It was therefore not possible for us to obtain time-course data by NMR. All six patients showed a strong signal at 36.9 ppm for succinate C2 and C3 (2,3-^13^C_2_ succinate). This is a singlet because succinate is a symmetrical molecule so the C2 and C3 positions are equivalent. The strongest metabolite signals were doublets (J = 37.3 Hz) for malate C3 at 45.2 ppm and C2 at 73.1 ppm, in all six patients, indicating 2,3-^13^C_2_ malate. There was no discernable singlet for malate. These results – malate doublets, but no singlets – indicate that the malate was entirely derived, without breaking the covalent bond between the two ^13^C atoms, from the 2,3-^13^C_2_ succinate administered. A singlet at 138.0 ppm for C2 and C3 of fumarate (indicating 2,3-^13^C_2_ fumarate) was seen in all six patients. This is a singlet because fumarate is a symmetrical molecule. Doublets (J = 36.8 Hz) for lactate C3 (methyl group) and for lactate C2 were observed in all six patients, at 22.8 and 71.3 ppm, respectively, indicating the presence of 2,3-^13^C_2_ lactate, together with singlets for lactate C3 and C2.

Doublets (J = 34.8 Hz) were observed at 57.4 and 30.4 ppm, representing glutamine C2 and C3, respectively, indicating the presence of 2,3-^13^C_2_ glutamine in five of six patients, together with singlets for glutamine C2 and C3. The remaining patient (TBI Patient 1) showed no clear doublet or singlet signals discernable above noise for Gln C2 or Gln C3. Peak identities were corroborated on NMR by two-dimensional ^1^H-^13^C HSQC spectra. Glutamine is synthesised from glutamate that is a spin-off from the TCA cycle intermediate alpha-ketoglutarate. The presence of doublets for glutamine C2 and C3 indicates metabolism of 2,3-^13^C_2_ succinate by the TCA cycle with the covalent bond between the two ^13^C atoms intact. No other doublet signals were observed for glutamine (or glutamate).

None of the six patients showed a clear glutamine C4 singlet above baseline noise. Quantification of the C2 and C3 glutamine singlets (after subtracting background natural abundance ^13^C) revealed small degrees of fractional enrichment with medians of 1.1% (0%–3.6%) and 3.5% (1.1%–6.9%), respectively.

Pyruvate was undetectable in our ^13^C NMR spectra. Pyruvate’s chemical shifts (C3 at 29.15 ppm, C2 at 207.7 ppm and C1 at 172.8 ppm, in spectra of standards) do not coincide with lactate or any other of our analytes. Pyruvate is at the crossroads of several biochemical pathways, being synthesised and consumed, so its concentration is never high, and microdialysis only samples the extracellular compartment. In the literature, ^13^C-labelled pyruvate is usually not itself detectable as a metabolite in NMR spectra of brain tissue extracts, and pyruvate’s involvement as an intermediate is inferred from detection of labelling in its products.^28,29^ Moreover, exogenous ^13^C-labelled pyruvate is very rapidly metabolised by brain.^30^

**Figure 5.**
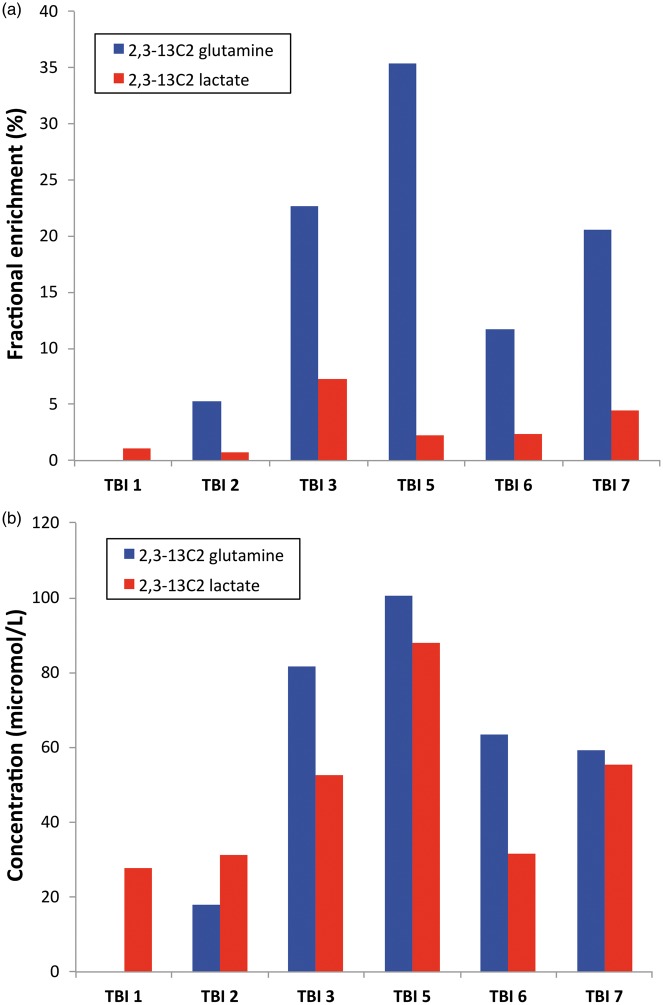
^13^C enrichment in glutamine and lactate. (a) Fractional enrichment values (%) for microdialysate 2,3-^13^C_2_ glutamine (blue bars; based on the glutamine C3 doublet signal) and 2,3-^13^C_2_ lactate (red bars; based on the lactate C3 doublet signal). (b) Corresponding concentrations (µmol/L) of 2,3-^13^C_2_ glutamine and 2,3-^13^C_2_ lactate.

Results for fractional enrichment (%) in 2,3-^13^C_2_ glutamine are shown in Figure 5(a). The median was 16.2% (5.3%–22.6%) measured using the C3 doublet. Similar fractional enrichment was seen for the glutamine C2 doublet, as expected. The ^13^C doublet signals at glutamine C2 and C3 were not background-subtracted because the probability of two natural endogenous ^13^C atoms occurring next to each other is 0.01%.

Fractional enrichment (%) results in 2,3-^13^C_2_ lactate are shown in Figure 5(a). Median (IQR) fractional enrichments of the lactate C3 and C2 doublets were 2.3% (1.1%–4.5%) and 2.0% (0.8%–4.4%).

The corresponding concentrations (in µmol/L) of 2,3-^13^C_2_ glutamine and 2,3-^13^C_2_ lactate are shown in Figure 5(b). Singlets for lactate C3 and C2 revealed no significant ^13^C enrichment above background natural abundance ^13^C. In five of six patients, enrichment was greater in 2,3-^13^C_2_ glutamine than in 2,3-^13^C_2_ lactate, while in the remaining patient (TBI Patient 1), 2,3-^13^C_2_ glutamine was undetectable although 2,3-^13^C_2_ lactate was present. The median (IQR) 2,3-^13^C_2_ lactate/2,3-^13^C_2_ glutamine enrichment ratio was 19.9% (12.9%–21.7%).

The ^13^C-labelling patterns identified are thus clear evidence for the metabolic pathways shown in Figure 1.

## Discussion

We have shown that 2,3-^13^C_2_ succinate (disodium salt) perfused via microdialysis catheters in TBI patients entered the TCA cycle evidenced by ^13^C-labelling patterns in metabolites (Figures 1 and 4), and also potentiated several aspects of brain chemistry: importantly decreases in extracellular LPR and glutamate concentration.

### Significance of double labelling patterns in metabolites

The characteristic NMR doublets arising from two adjacent ^13^C atoms prove cellular uptake and mitochondrial metabolism of 2,3-^13^C_2_ succinate revealing downstream metabolites. Doublets are clearly distinct from native endogenous molecules, because the probability of two endogenous ^13^C atoms being adjacent to each other naturally is 0.01% (a 1 in 10,000 chance) and indistinguishable from baseline ‘noise’ in these spectra. As SDH is exclusively mitochondrial, downstream metabolites of 2,3-^13^C_2_ succinate must be derived from pathways stemming from mitochondrial metabolism. In microdialysates, the extracellular concentrations and ^13^C labelling patterns allow deduction of cellular metabolic pathways. The doubly ^13^C-labelled metabolites are unambiguous evidence the 2,3-^13^C_2_ succinate molecules crossed from the perfusate into the brain extracellular space, entered the cells and were metabolised, exported into the extracellular fluid and recovered by the microdialysis catheter.

^13^C NMR clearly demonstrated that 2,3-^13^C_2_ malate, 2,3-^13^C_2_ glutamine and 2,3-^13^C_2_ lactate were among the metabolites, indicating TCA cycle metabolism with the C2-C3 carbon–carbon bond intact (Figure 5). 2,3-^13^C_2_ glutamine is a TCA spinout product originating from alpha-ketoglutarate, a TCA cycle intermediate exiting as glutamate, enzymatically inter-convertible with glutamine that predominates extracellularly.

The TCA cycle intermediate OAA can spin out aspartate, which can exit mitochondria (via the glutamate/aspartate carrier) via the malate-aspartate shuttle mechanism.^20^ In the cytosol, aspartate is converted to OAA and thence to malate, which can re-enter mitochondria (via the malate/alpha-ketoglutarate carrier). The 2,3-^13^C_2_ malate detected in microdialysates may have emerged from the TCA cycle via this pathway.

Our identification of 2,3-^13^C_2_ lactate among the metabolites of 2,3-^13^C_2_ succinate is the first demonstration of the TCA cycle spin-out route of lactate production in human TBI brain, and merits investigation. Previous indications were in animal brains.^28,29,31^ The TCA cycle intermediates malate and OAA can be converted to pyruvate by malic enzyme (ME); also OAA can be converted by phosphoenolpyruvate carboxykinase (PEPCK) plus pyruvate kinase (PK) to pyruvate, which LDH can convert to lactate. Our finding of TCA cycle-derived lactate may have implications for interpreting high extracellular levels of lactate and LPR, hitherto simply attributed to glycolysis. An LPR > 25 is interpreted as either hypoxia or ‘mitochondrial dysfunction’. The latter remains poorly understood, and this study raises questions of whether the lactate (and implicitly pyruvate) derived from TCA-cycle spin-out, termed cataplerosis^31^ forms a significant proportion of total extracellular lactate (and pyruvate), whether cataplerotic levels change in injured vs. normal brain, and biological implications.

### Pyruvate recycling – Partial versus full

Lactate generation by TCA cycle cataplerosis is also termed ‘partial pyruvate recycling’, as the pyruvate spun out from the TCA cycle does not re-enter it but proceeds to lactate that exits from cells.^31^ This appears distinct from full pyruvate recycling^28,32,33^ whereby spun-out pyruvate is converted by pyruvate dehydrogenase (PDH) to acetate that re-enters the TCA cycle. We found no evidence for full recycling, as spun-out 2,3-^13^C_2_ pyruvate conversion by PDH to 1,2-^13^C_2_ acetate entering the TCA cycle would have produced 4,5-^13^C_2_ glutamine,^32^ which we did not detect. Neither did we detect 1,2-^13^C_2_ lactate, which would have resulted from subsequent spin-out from 1,2-^13^C_2_ oxaloacetate, and there was no significant enrichment for 3-^13^C lactate, which would have arisen from spin-out from 3,4-^13^C_2_ oxaloacetate.

Re-entry of 2,3-^13^C_2_ pyruvate via anaplerotic pathways pyruvate carboxylase (PC) or ME into the TCA cycle would produce 2,3-^13^C_2_ glutamine, the same ^13^C-pattern as if 2,3-^13^C_2_ succinate metabolism proceeds directly (without exit of intermediates and re-entry) round the TCA cycle to 2,3-^13^C_2_ alpha-ketoglutarate and spins out 2,3-^13^C_2_ glutamine. Re-entry via PC or ME seems unlikely in the absence of evidence for re-entry via PDH. Therefore, the 2,3-^13^C_2_ glutamine detected is probably from 2,3-^13^C_2_ succinate metabolism proceeding directly round the TCA cycle, further supported by fractional enrichment (%) for ^13^C being greater (in five of six patients) in 2,3-^13^C_2_ glutamine than in 2,3-^13^C_2_ lactate (Figure 5(a)).

### Succinate perfusion potentiates local brain chemistry

Conventional measures (regardless of labelling), on an ISCUS microdialysis analyser, at baseline and during 2,3-^13^C_2_ succinate perfusion, showed glucose concentration decrease suggesting greater utilisation, a lower LPR due to relatively raised pyruvate, and glutamate decrease. LPR ‘Type 2’ elevation characterised by low pyruvate without hypoxia or ischaemia is attributed to metabolic disturbance.^34^ Increasing pyruvate and thereby lowering LPR thus concurs with better brain metabolism, consistent with TCA cycle operation indicated by the ^13^C-labelling patterns.

To generate 1 mole of pyruvate by glycolysis, 1 mole of NAD^+^ is converted into NADH, which must be recycled (oxidised) back to NAD^+^ to sustain glycolysis. Recycling can occur by the mitochondrial ETCs (if operational). NADH cannot cross the mitochondrial membrane, so hydrogens and electrons are transferred indirectly by shuttles (malate-aspartate and/or glycerol 3-phosphate).^20^ Malate-aspartate shuttle operation concurs with 2,3-^13^C_2_ malate in microdialysates. In mitochondrial dysfunction or hypoxia, NADH can be recycled to NAD^+^ by LDH-mediated conversion of pyruvate to lactate in the cytosol, producing a high extracellular LPR.

Lowering of brain extracellular glucose during succinate perfusion, suggesting improved glucose utilisation, together with a lower extracellular LPR, may stem from better redox behaviour by aforementioned NADH to NAD^+^ recycling by mitochondrial shuttles,^20^ lessening LDH-mediated conversion of pyruvate to lactate. The lower extracellular glutamate during succinate perfusion may result from the malate-aspartate shuttle, drawing glutamate into mitochondria for consumption via the TCA cycle, preventing excitotoxic glutamate build-up. High extracellular glutamate and glucose levels are associated with adverse clinical outcomes.^3^

Conventionally, the microdialysis literature ascribes glycerol increase to phospholipid degradation suggesting cell death.^35^ However, debate exists^34–36^ because glycerol is a glucose metabolite^37–39^ and glycerol increase during succinate perfusion may arise from increased glycolysis. Glycerol decreased after succinate perfusion ceased, in the two patients whose baseline period was post-succinate (Figure 3), concurring with metabolically derived glycerol, rather than cell death. Conceivably, glycerol may increase by succinate fuelling the mitochondrial ETC enhancing the glycerol 3-phosphate (G3P) shuttle, recycling NADH to NAD^+^ in the cytosol when dihydroxyacetone phosphate (DHAP; a glycolysis intermediate) is reduced by cytosolic glycerol 3-phosphate dehydrogenase (GPDH) to G3P.^20^ This is re-oxidized to DHAP by mitochondrial GPDH and electrons transferred to CoQ and enter the mitochondrial ETC.^20^ Like the malate-aspartate shuttle (above), enhancing the G3P shuttle would improve recycling of NADH to NAD^+^ by mitochondrial ETC, giving lower LPR and more efficient glucose utilisation.

### Importance of oxygen

PbtO_2_ measurements indicated oxygen supply to the region that received disodium succinate. Without oxygen, the mitochondrial ETC cannot function properly. In experimental ischaemia, SDH (ETC complex II and part of the TCA cycle) ran ‘backwards’, building up succinate and reverse electron transport to complex I.^40^ Reperfusion produced a surge in reactive oxygen species (via complex I) and cell death.^40^ Dimethyl succinate (an artificial, lipophilic derivative much more taken up by cells than disodium succinate) exacerbated ischaemia–reperfusion damage, while malonate, a TCA cycle inhibitor that blocks SDH, protected against ischaemia–reperfusion injury.^40^ Our present study did not involve ischaemia–reperfusion. We suggest that using succinate to support mitochondrial metabolism should be performed in the presence of adequate oxygenation, not ischaemia–reperfusion.

In circumstances like our study, where oxygen appears not limiting, oxidative phosphorylation is limited by mitochondrial efficiency, which appears improved (lower microdialysate LPR) by succinate administration. The doubly ^13^C-labelled metabolites unambiguously prove that the disodium 2,3-^13^C_2_ succinate was taken up and metabolised by mitochondria.

### Limitations

The reason we combined brain microdialysates for 24 h per patient was to provide enough material for ^13^C NMR within a workable spectrum acquisition time on the NMR spectrometer. Our 500 MHz NMR spectrometer has a cryoprobe^21,22^ that is more sensitive than non-cryo probe technology, but, nevertheless, limits how short a microdialysis timeframe we can analyse. NMR micro-cryoprobes are commercially available, for smaller samples, and future adoption, with higher-field NMR spectrometers for even greater sensitivity, if accessible, will improve time-resolution for tracking biochemical pathways. We did not analyse blood. Focal brain micro-dosing is unlikely to yield measurable labelling in blood, due to dilution of ^13^C-labelled species with unlabelled endogenous species and physical volume of blood. Neurocritical care protocol-driven therapy includes maintaining a serum glucose 4–7 mmol/L target range. While serum glucose and glycaemic control can influence brain glucose, this relationship is not necessarily straightforward and may be lost in injured brain,^27,41^ meriting dedicated study with continuous monitoring of both blood and brain.

Cerebral microdialysis is an invasive technique and is thus effectively limited to patients being monitored following severe brain injury. These patients are sedated (see ‘Patients and methods’ section), so there is no placebo effect – they are unconscious. On account of its invasive nature, there are no truly normal controls for cerebral microdialysis in humans. It has sometimes been possible to perform microdialysis in radiologically normal brain areas in patients undergoing surgery for removal of benign tumours in a different part of the brain,^22,42^ but patient considerations and practical matters make such sampling rare. In the present study, we used each TBI patient as his or her own control (see ‘Patients and methods’ and ‘Results’ sections). In further support of this approach, it has been noted in the literature that microdialysis data are highly auto-correlated even at up to a future 30 h, and subject identity alone explains 52% to 75% of microdialysis marker variance, determined in a large study (comprising more than 7350 hourly samples of complete microdialysis sets from 90 TBI patients) using statistical and pattern-recognition computer models.^24^ Thus, microdialysate data are highly individualised and it was therefore logical to use each patient as his/her own control rather than comparing with a separate control group of different TBI patients.

Mitochondrial dysfunction is defined as an inability to generate energy despite ‘adequate’ provision of metabolic fuels and oxygen. This suggests a fundamental biochemical failure at the mitochondrial level following TBI. Mitochondrial dysfunction is undoubtedly a complex phenotype and exploring its full ramifications is outside the scope of the present study. We have established that TBI brain retains sufficient mitochondrial capability to metabolise exogenous succinate by the TCA cycle, proved using double ^13^C-labelling. This exogenous fuelling of the TCA cycle resulted in apparent improvements in brain chemistry judged by surrogate endpoints including LPR decrease. Whether such changes would translate to better clinical outcome would need a more extensive, larger study in TBI patients. Further support for benefits of augmenting brain mitochondrial oxidative metabolism comes from studies in rats with methylene blue,^43^ or with kaempferol,^44^ which enhances mitochondrial matrix uptake of Ca^2+^ and boosts oxidative metabolism, which led to improved brain function and behavioural outcomes after experimental brain injury in a rat TBI model.^45^

## Conclusion

Here we have shown that 2,3-^13^C_2_ succinate delivered by microdialysis can interrogate the TCA cycle’s role in producing ^13^C-labelled metabolites exported into the interstitium, proof-of-concept that the TCA cycle can be directly supplemented and TBI brain chemistry potentiated. Our novel discovery of the TCA cycle as a source of lactate in human TBI brain calls for revision of lactate’s significance, hitherto regarded as simply glycolytic, with possible implications for other diseases (e.g. cancer). Lower LPR suggests that succinate improves redox balance, conceivably by boosting shuttles utilising mitochondrial ETCs to recycle NAPH to NAD^+^, possibly promoting glucose utilisation and glutamate clearance from the interstitium. Whether such metabolic shifts would ameliorate ATP generation merits investigation. Specific intervention that modifies the LPR provides a promising future avenue.^27^ This study opens the prospect of enhancing mitochondrial metabolism by using TCA cycle products/substrates such as succinate or other species as a potential therapeutic strategy, for brain injury and other neurological disorders involving mitochondrial dysfunction.

## Funding

The author(s) disclosed receipt of the following financial support for the research, authorship, and/or publication of this article: Medical Research Council (Grant Nos. G0600986 ID79068 and G1002277 ID98489) and National Institute for Health Research Biomedical Research Centre, Cambridge (Neuroscience Theme; Brain Injury and Repair Theme). Authors’ support: IJ – Medical Research Council (Grant no. G1002277 ID 98489) and National Institute for Health Research Biomedical Research Centre, Cambridge; KLHC – National Institute for Health Research Biomedical Research Centre, Cambridge (Neuroscience Theme; Brain Injury and Repair Theme); CG – the Canadian Institute of Health Research; AH – Medical Research Council/Royal College of Surgeons of England Clinical Research Training Fellowship (Grant no. G0802251) and Raymond and Beverly Sackler Fellowship; DKM and JDP – National Institute for Health Research Senior Investigator Awards; PJH – National Institute for Health Research Professorship, Academy of Medical Sciences/Health Foundation Senior Surgical Scientist Fellowship and the National Institute for Health Research Biomedical Research Centre, Cambridge.

## Supplementary Material

Supplementary material
