# Supplementary material for: Focally perfused succinate potentiates brain metabolism in head injury patients
Source: J Cereb Blood Flow Metab. 2016 Jan 1;37(7):2626–38. doi: 10.1177/0271678X16672665 (PMC5482384; doi:10.1177/0271678X16672665)
Supplement: Supplementary material [file SupplementaryTable_2665.pdf]

Supplementary Table 1. ISCUS clinical microdialysis analyser measurements. Results are medians during 24 h baseline perfusion (with plain unsupplemented CNS perfusion fluid) and during 24 h perfusion with 2,3-$^{13}C_2$ succinate (disodium salt; 12 mmol/L).

TBI Patient ID	Glc base mM	Glc succ mM	LPR base ratio	LPR succ ratio	Lac base mM	Lac succ mM	Pyr base µM	Pyr Succ µM	Gly base µM	Gly succ µM	Glt base µM	Glt succ µM
1	2.35	2.62	19.5	18.7	3.93	3.37	204.16	177.47	84.20	349.92	--	19.36
2	4.24	2.89	17.4	16.2	4.71	5.24	261.45	329.42	99.09	197.93	4.60	2.07
3	3.77	2.73	10.7	9.4	1.03	0.85	87.23	89.75	16.18	--	--	--
4	3.25	2.51	19.5	13.1	2.49	2.38	144.57	182.51	191.87	149.86	2.40	2.07
5	3.38	2.48	22.9	21.0	4.09	4.71	178.42	227.94	35.60	53.52	14.82	4.28
6	1.79	1.49	13.8	14.7	1.33	1.76	93.54	123.81	38.86	125.70	6.60	3.22
7	1.47	1.57	16.9	15.8	1.28	1.64	75.26	102.45	29.53	391.59	1.95	1.63
8	1.16	0.99	31.3	21.5	8.36	7.90	267.05	360.21	88.65	--	12.88	8.93
9	0.95	0.82	26.8	23.29	7.20	4.85	275.22	222.76	--	--	12.63	4.91

Abbreviations and footnotes: Glc, glucose; LPR, lactate/pyruvate ratio; Lac, lactate; Pyr, pyruvate; Gly, glycerol; Glt, glutamate. "Base" indicates baseline concentrations obtained with plain CNS perfusion fluid and "succ" indicates concentrations obtained with 2,3-$^{13}C_2$ succinate supplemented perfusion fluid. -- indicates not measured. These results are graphed in Fig 3. For Patients 1 and 2 the baseline period was post-succinate, while for the other seven patients the baseline period was pre-succinate. Changes between baseline and succinate perfusion were significant for lactate/pyruvate ratio ($p = 0.0152$), glucose ($p = 0.038$) and glutamate ($p = 0.018$) by pair-wise nonparametric statistical analysis (Wilcoxon's signed rank test).
